# Mapping the neglected topic in head and neck paraganglioma research: a PRISMA scoping review on quality of life

**DOI:** 10.1007/s11136-026-04218-8

**Published:** 2026-03-13

**Authors:** Djenghiz P. S. Samlal, Marina Hristova, Angelika Albrecht, Joerg Schipper, Thijs T. G. Jansen, Corinne P. A. Delsing, Henricus P. M. Kunst

**Affiliations:** 1 Department of Otolaryngology and Head and Neck Surgery, Radboud University Medical Center, Nijmegen, The Netherlands; 2 Dutch Academic Alliance Skull Base Pathology, Radboud University Medical Center, Maastricht University Medical Center+, Nijmegen, Maastricht, The Netherlands; 3 Department of Otolaryngology and Head and Neck Surgery, Heinrich Heine University Düsseldorf, Düsseldorf, Germany; 4 Department of Otolaryngology and Head and Neck Surgery, Maastricht University Medical Center+, Maastricht, The Netherlands

**Keywords:** Paraganglioma, Carotid body, Jugulotympanic, Quality of life, Surgery, Radiotherapy

## Abstract

**Purpose:**

Paragangliomas in the head and neck (HNPGL) are rare slow growing neuro-endocrine tumors. The impact of various treatment strategies and tumor characteristics on health-related quality of life (HRQoL) remains unclear. This scoping review aims to map the available evidence and summarize the findings on how HNPGL affects patients’ HRQoL.

**Methods:**

A comprehensive literature search was conducted in PubMed, Embase, and Cochrane. Articles were included when presenting a quantification of HRQoL or Patient Reported Outcome Measures in patients with HNPGL (*n* ≥ 5). Studies focusing exclusively on paragangliomas outside the head and neck region were excluded. A scoping review using descriptive and inductive thematic analysis was performed and presented using the Prisma-ScR guidelines.

**Results:**

Fourteen studies were included presenting quantifiable HRQoL data in patients with HNPGL. The most used HRQoL measure were the Short Form-36 (*n* = 4, 29%) and EORTC Core Quality of Life questionnaire (QLQ-C30) (*n* = 3, 21%), followed by Short Form-12 (*n* = 2, 14%). HNPGL negatively affects HRQoL across several domains, even in patients not requiring an intervention. Specific subgroups, including those with carotid body tumors, multiple tumors, or dysphonia, reported disproportionately lower HRQoL.

**Conclusion:**

Current sparse evidence on HRQoL in patients with HNPGL demonstrates significant impairment and identified various factors influencing this outcome. However, further research is necessary to specifically assess the effect of different therapeutic managements on HRQoL.

**Supplementary Information:**

The online version contains supplementary material available at 10.1007/s11136-026-04218-8.

## Plain English Summary

Paragangliomas are rare tumors that can grow in the head and neck region. Although most do not spread throughout the body and grow slow, they may press on important nerves and blood vessels, potentially requiring surgery or radiation. For many patients, it is unclear whether treatment will affect their overall well-being and quality of life. This review helps clarify that question. We systematically searched the scientific literature for studies that measured quality of life in patients with head and neck paragangliomas (HNPGL). We included 14 studies that used questionnaires such as the SF-36 and EORTC QLQ-C30 to assess physical, emotional, and social functioning. We found that HNPGL negatively impacts quality of life in several ways—even in patients who do not undergo treatment. Patients with tumors in specific locations (e.g., carotid body), multiple tumors, or difficulties speaking reported lower quality of life. However, evidence is limited, especially about the effects of treatment over time. In summary, this review shows that quality of life is often reduced in people with HNPGL. We highlight the need for more studies that follow patients before and after treatment and for better tools to measure their well-being over time.

## Introduction

Paragangliomas are rare, slow growing, highly vascularized neuroendocrine tumors with variable metastatic potential originating from extra-adrenal parasympathetic and sympathetic paraganglia. With an estimated overall incidence of 0.3–1 per 100.000, paragangliomas in the head and neck (HNPGL) encompass 65% to 70% of all paragangliomas [1–3], 0.6% of head and neck tumors, and 0.03% of all tumors [3, 4]. HNPGL are frequently located at the carotid bifurcation (60%), jugular bulb (30%), and vagal nerve (10%) [5]; sporadically reported are sinonasal, laryngeal, and cervical sympathetic chain paraganglioma [6–9]. Development of these tumors are increasingly linked to genetic variants of the succinate dehydrogenase genes (SDHx). Patients who inherit these autosomal dominant HNPGL-syndromes, therefore require lifelong surveillance [10], which may itself influence a patients’ quality of life (QoL). Metastasis occur in 9% to 13% of HNPGL and are independently associated with a younger age at diagnosis, genetic variants in SDHB gene, larger tumor size, and HNPGL genetic in the carotid body [10]. Clinical presentation varies from an asymptomatic neck mass to life-threatening tumors, depending on affected structures. Among other things, this can lead to cranial nerve paralyses or dysphagia [11, 12].

A multidisciplinary therapeutic management strategy includes active surveillance, radiotherapy, and (complete or subtotal) resection depending on tumor characteristics, specific genetic variants, and/or residual cranial nerve function [3, 10, 13, 14]. Historically, HNPGL have been primarily treated with surgical resection [15]. However, since most HNPGL grow slowly, more conservative options are increasingly considered in order to reduce the risk of cranial nerve injury and operative morbidity [11, 16, 17]. Although the survival of HNPGL patients has not been extensively studied, it appears that Dutch patients (*n* = 86) even after 50 years of follow up do not show any significantly reduced life expectancy when corrected for surgical mortality (*p* = 0.998) [18]. The management of HNPGL should thus also focus on improving or maintaining QoL [3, 19]. Frequently used functional outcomes (e.g., cranial nerve deficit or residual hearing) are valuable in their status as predictor for or proxy of the patients’ QoL. However, QoL can also be measured directly, leading to better understanding of the impact of proposed treatments for individual patients.

Previous HNPGL reviews have focused on outcomes such as local tumor control or volume, intraoperative blood loss, complications, hearing loss, dysphagia, lower cranial nerve function, however none aimed to take the QoL of patients with HNPGL into account [17, 20–25]. Health-related quality of life (HRQoL) is a multidimensional assessment of how disease and treatment affect a patient’s sense of overall functioning and well-being [26]. When answered by the patient themselves, questionnaires capture the experience and impact of impairments or limitations instead of merely its attributes. These patients reported outcome measures (PROMs) range from generic to disease-specific HRQoL or even symptom-specific questionnaires. Generic HRQoL measures provide the ability to compare the impact with other conditions, while disease-specific measures may be more sensitive for the detection and quantification of small changes that are important to clinicians or patients [27]. To date, no disease specific HRQoL measure has been developed for patients with HNPGL.

The goal of this study is to systematically map the research regarding the HRQoL of patients with HNPGL and identify existing gaps in knowledge. A scoping review according to Levac et al. provides a structured framework to clarify this complex concept and refine subsequent inquiries; it is particularly suited for disciplines with emerging evidence and paucity of controlled trials [28]. The following research questions were formulated: (1) Which methods and questionnaires are used to quantify HRQoL in patients with HNPGL? (2) What is known about the impact on HRQoL of the various HNPGL subtypes and interventions?

## Methods

This study followed the ethical guidelines of the Radboud University Medical Center, the Netherlands. A scoping review was conducted according Levac et al. [28] and reported according to PRISMA-ScR [29]. A research protocol was developed before the start of the review and registered in our tertiary care center’s database.

A team consisting of the authors and the Dutch Society for Patients with Paraganglioma identified the previously mentioned research questions. A comprehensive systematic search was performed using databases PubMed, Embase, and Cochrane on 07-10-2024, and updated on 24-05-25. The search string per database is supplied in Supplement 1; it combined synonyms of various HNPGLs with either generic synonyms for HRQoL and PROMs or specific questionnaires. Questionnaires resulting from post-hoc analysis were added to the search. Backward citation searching was conducted to identify relevant articles.

Articles were included when presenting quantified HRQoL or PROM data in patients with HNPGL, had a cohort size ≥ 5, and full text was available. For inclusion in sub-analyses, HRQoL needed to be stratified by either genetic variant, treatment modality, tumor location, or tumor classification. Studies were excluded when only paraganglioma outside the head and neck region were included in the data. No filters based on publication date or language was used.

Manual de-duplication, title and abstracts screening in Endnote21, data-extraction in Excel 2021, and quality assessment was performed independently by two reviewers (DS and HK). Full texts were assessed for eligibility based on in-/exclusion criteria. No AI-based decision making was used. The authors of the included studies were not contacted to obtain additional information or clarification beyond what was reported in the published manuscripts. Although critical appraisal is not a prerequisite in a scoping review, the validated Joanna Briggs Institute’s critical appraisal tool for cross-sectional studies was used to inform the interpretation of findings and identify methodological limitations [30]. Critical appraisal, data extraction and charting including the HRQoL measurement methodology, studied population, interventions, and outcomes was performed independently by two reviewers (DS and HK). Discrepancies between the two reviewers were resolved by discussion. An iterative data extraction process according to Levac et al. was conducted to include relevant parameters for stratification [28]. When the original data was not stratified but predominantly reflected one subgroup, results were attributed accordingly. The HRQoL measures were descriptively summarized, and content analysis was presented based on inductive thematic analysis.

## Results

### Study selection and characteristics

**Fig. 1:**
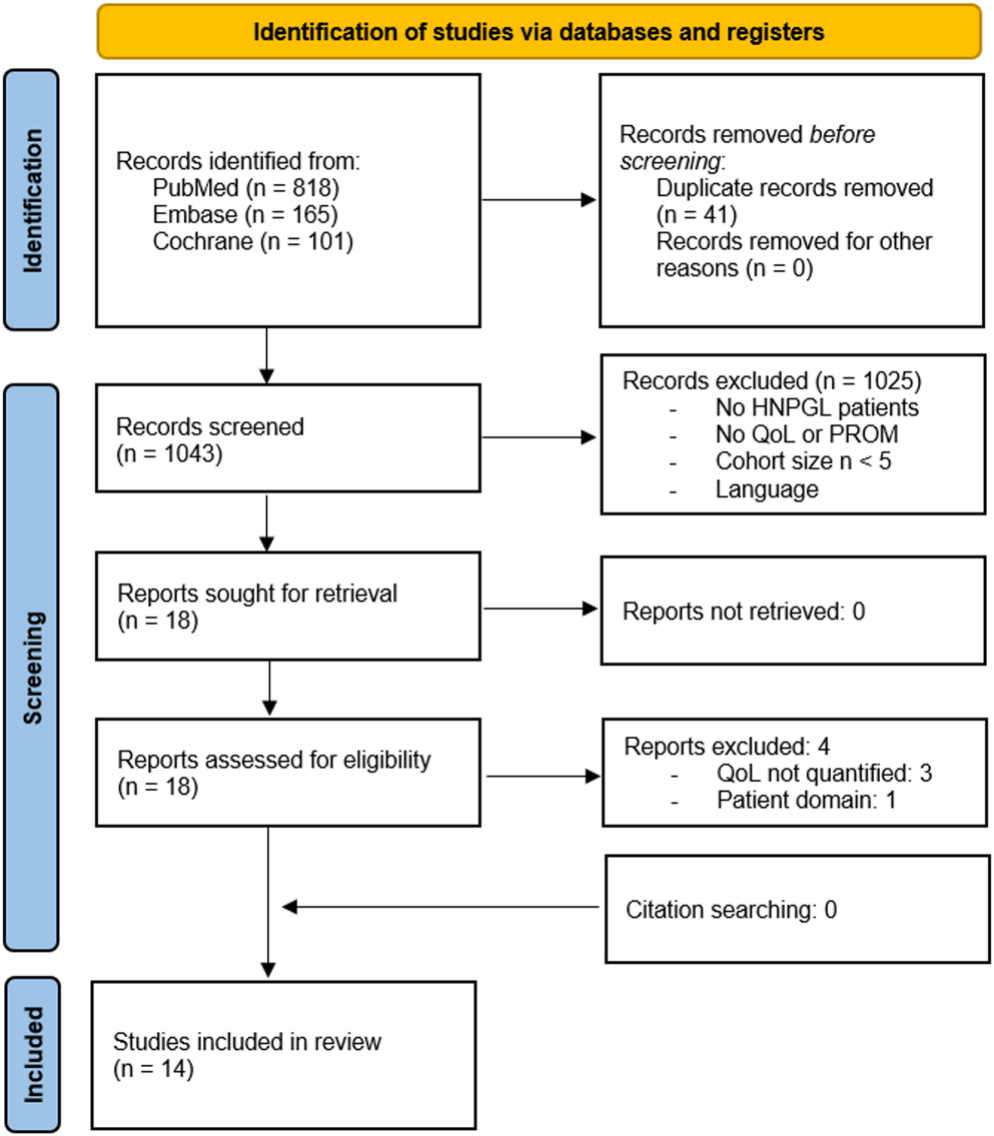
Study selection PRISMA flowchart

Screening of 1043 unique records resulted in 14 included studies published between 1998 and 2025 [19, 31–43]. Citation searching of included and key records identified no additional results (Fig. 1).

Studies originated predominantly from Europe, particularly the Netherlands (36%), Germany (21%), and France (14%). Most studies were published in the past 15 years (71%). Table 1 presents the characteristics of the included studies.

Six studies (42%) focused on jugulotympanic paraganglioma (GJT), two studies (14%) on carotid body tumors (CBT), while the remaining (42%) did not differentiate the HRQoL measures by HNPGL tumor location. Van Hulsteijn et al. (2013) included twenty-eight out of their 174 (16%) patients with paragangliomas only located outside the head and neck. De Bresser et al. (2025) did not specify the location(s) of paragangliomas; however, their cohort is said to be selected from a register that focusses on HNPGL. Two studies included HRQoL measures for SDHx genetic variant carriers without manifestation of paragangliomas. One study stratified outcomes based SDHx genetic variants. Seven studies (50%) provided HRQoL of untreated patients, seven (50%) after surgery, five (36%) after radiotherapy, and one after proton therapy (7%). Two studies (14%) solely focused on sleep related problems.

No randomized controlled trials were identified. Three studies were prospective (21%), while the majority of studies identified their subjects retrospectively. A control group was present in only four studies (29%). Cohort sizes varied from 8 to 174 patients. Given the variations in study design, reporting quality, presented subgroups, and outcome measures quantitative synthesis and meta-analysis of the results was not possible.

**Table 1:** Study characteristics

Study	Year	Country	Subject identification	Cohort period	QoL measured / total cohort	Tumor location	Therapy	QoL/PROM tool	Timing
Brinner et al.	1998	Switzerland	Retrospective	1981–1986	36 / 50	GJT	Surgery	Self-designed questionnaire	cross-sectional
Kollert et al.	2006	Germany	Retrospective	1981–2004	29 / 79	HNPGL	Surgery	SIP	cross-sectional
Henzel et al.	2007	Germany	Prospective	1999–2005	17 / 17	GJT	RTx	SF-36	12mo after RTx
Havekes et al.	2008	Netherlands	Retrospective	N/A	82 / 105	HNPGL	No, Surgery	SF-36, HADS, MFI-20, NHP	cross-sectional
Havekes et al.	2012	Netherlands	Retrospective	N/A	74 / 105	HNPGL	No, Surgery	ESS, SGRQ, SCA	cross-sectional
Van Hulsteijn et al.	2013	Netherlands	Retrospective	N/A	174 / 302	HNPGL or carrier	No, Surgery	SF-36, HADS, MFI-20	cross-sectional
Van Hulsteijn et al.	2014	Netherlands	Prospective	N/A	16 / 16	CBT	No	ESS, MFI-20	cross-sectional
Galland-Girodet et al.	2014	France	Retrospective	1987–2010	20 / 30	HNPGL	RTx, RTx+ Surgery	EORTC QLQ-C30 + H&N35	cross-sectional
Cao et al.	2018	France	Retrospective	2000–2014	8 / 10	GJT	Proton therapy	EORTC QLQ-C30 + H&N35	cross-sectional
Garcia-Alva et al.	2019	Mexico	Retrospective	2017–2018	23 / 24	CBT	No	SF-36	cross-sectional
Patel et al.	2019	USA	Retrospective	1990–2017	26 / 69	GJT	RTx	SF-36, PROMIS-10, GBI, EAT-10, VHI, VRQoL	cross-sectional
Ehret et al.	2020	Germany	Prospective	2005–2018	35 / 53	GJT	No, RTx	SF-12	longitudinal
Hebb et al.	2020	Canada	Retrospective	N/A	23 / 30	GJT	No, RTx, RTx+ Surgery	SF-12, HHIA, DHI, THI	cross-sectional
De Bresser et al.	2025	Netherlands	Retrospective	2022–2023	86 / 100	HNPGL or carrier	No, Surgery	EORTC QLQ-C30, EQ-5D-5 L, CWS, HADS, MFI-20	cross-sectional

CBT, carotid body tumor; CWS, cancer worry scale; DHi, dizziness handicap inventory; EAT-10, eating assessment tool-10; EQ-5D-5 L, EuroQol 5D-5 L; ESS, epworth sleepiness scale; GBI, glasgow benefit inventory; GJT, jugulotympanic paraganglioma; HADS, hospital anxiety and depression scale; HHIA, hearing handicap inventory for adults; HNPGL, head and neck paraganglioma not specified; MFI-20, multidimensional fatigue index; N/A, not available; NHP, Nottingham health profile; SCA, standardized clinical assessment of sleep related complaints; SF-36, Short Form 36; SGRQ, St. George respiratory questionnaire; SIP, Sickness impact profile; THI, tinnitus handicap inventory; VHI, voice handicap index; VRQoL, voice related quality of life

### Questionnaires used for quantifying HRQoL in patients with HNPGL

No HNPGL disease-specific questionnaires were identified. The most used generic HRQoL measure was the Short Form-36 (SF-36) (*n* = 4, 29%) followed by the European Organisation for Research and Treatment of Cancer (EORTC) Core Quality of Life questionnaire (QLQ-C30) (*n* = 3, 21%), and Short Form-12 (SF-12) (*n* = 2, 14%). Sickness Impact Profile and a self-designed questionnaire were used once each (7%). Depending on the questionnaire results were stratified per domain.

The Multidimensional Fatigue Index (MFI-20) (*n* = 4, 29%) and Hospital Anxiety and Depression Scale (HADS) (*n* = 3, 21%) were the most administered PROMs. The remaining PROMs are shown in Table 1; they concerned sleep problems, respiratory function, and various cranial nerves’ function.

### Effects of SDHx genetic variants and untreated HNPGLs

No statistically significant difference in HRQoL scores (SF-36, HADS, MFI-20) was observed when SDHx genetic variant carriers without tumors (*n* = 25) was compared to self-provided controls or age-adjusted reference values [35]. Patients with hereditary HNPGL had similar HRQoL compared to those with a sporadic HNPGL [35, 43].

HRQoL of untreated HNPGL patients is measured in 5 studies using either the SF-36 or SF-12 [33, 35, 37, 39, 40]. All except Hebb et al. (2020) provided aggregated scores per short form domain which are collected in Table 2. All studies with a control group showed a statistically significant decrease in HRQoL across some domain(s) compared to their own controls. No differences in anxiety or depression were found between patients and controls using the HADS questionnaire [33, 35].

**Table 2:** Mean HRQoL measured with SF-36 or SF-12 in untreated HNPGL patients

Study	Havekes 2008	van Hulsteijn 2013	Garcia-Alva 2019	Ehret 2020	Age adjusted reference values [44]	Vestibular Schwannoma [45]
Population	HNPGL (*n* = 82)	HNPGL (*n* = 146)^a^	CBT (*n* = 23)	GJT (*n* = 35)	Norwegian population sample^b^	Untreated vestibular Schwannoma (*n* = 130)
Timing	Cross-sectional	Cross-sectional	Before surgery	Before RTx		
Own control group	Patients provided case-control	Patients provided case-control	Case-control	N/A		
Physical functioning	86.7 ± 17.2 *	84.0 ± 18.9 *	80 (20–100) *	68.5 ± 31.7	87.9 ± 16.1	77.3 ± 24.1
Role limitations (physical)	73.2 ± 36.8	68.5 ± 41.0 *	75 (0–100) *	64.2 ± 28.7	73.4 ± 39.3	64.3 ± 40.4
Bodily pain	83.1 ± 19.5 *	82.6 ± 21.8	72 (22–100) *	71.4 ± 30.4	66.4 ± 25.5	75.3 ± 26.5
General health perceptions	66.8 ± 21.8 *	62.7 ± 22.9 *	56 (10–86) *	48.8 ± 26.7	72.8 ± 22.8	60.2 ± 20.2
Energy/vitality	N/A	60.9 ± 20.2 *	60 (20–100) *	52.1 ± 29.3	56.1 ± 20.8	63.6 ± 22.2
Social functioning	82.8 ± 23.1 *	78.0 ± 24.3 *	75 (37–100) *	65.7 ± 28.5	85.1 ± 21.3	70.7 ± 26.9
Role limitations (emotional)	81.7 ± 34.8 *	79.7 35.4	66 (0–100) *	65.7 ± 26.3	91.6 ± 22.0	77.6 ± 34.6
Mental health	N/A	N/A	60 (20–92) *	59.2 ± 22.5	80.2 ± 13.8	72.0 ± 19.7

(…), Range; ±, Standard deviation; CBT, Carotid Body Tumor; GJT, Jugulotympanic paraganglioma; HNPGL, Head and Neck Paraganglioma not specified; N/A, not available; SF-36/12, Short Form 36/12.

^a^ Cohort included HNPGL (*n* = 146) and paragangliomas outside the head and neck (*n* = 28).

^b^ Reference values for females aged 50–59 based on baseline characteristics of included studies (*n* = 260).

*Statistically significant difference compared to own control group.

### Tumor specific HRQoL and the effect of interventions

An analysis on untreated CBT found no statistically significant difference between each quartile tumor volume groups (mean 27cm^3^, range 5–99) using the SF-36 [37]. However, the Shamblin classification showed significant differences in physical function and role limitation due to physical problems suggesting that patients with untreated Shamblin III have worse QoL than those with Shamblin II in the aforementioned domains regardless of tumor volume [37].

Self-reported sleep-related problems are significantly increased in HNPGL patients compared to age-adjusted controls [31, 32, 34]. CBT were associated with greater fatigue and increased sleep related complaints [33–36]. The presence of CBTs and dysphonia were independent predictors of daytime sleepiness [34]. Only in patients with bilateral CBT a non-significant trend was observed towards a higher apnea/hypopnea index (*p* = 0.06) compared to the control [36].

GJT were commonly studied in the context of radiotherapy [32, 38–40]. The SF-12 was unable to detect a statistically significant difference between HRQoL in patients with untreated GJT and age-adjusted refence values [39]. Furthermore, the SF-12 or SF-36 did not detect a statistically significant difference in those with irradiated HNPGL compared to age adjusted refence values [32, 38–40]. However, the EORTC QLQ-C30 did show a significant decreased HRQoL in patients with HNPGL who received either only RTx (*n* = 9) or surgery prior to RTx (*n* = 11) when compared to control patients from the EORTC Head and Neck Database (Table 3). All five functional scales were worse for these treated HNPGL patients [19]. Patients in the combined treatment group had, although they were younger (45 vs. 66 years, *p* = 0.014), a poorer HRQoL than those of the RTx only group (*p* = 0.01) and a greater (negative) impact of head and neck related symptoms [19].

No study presented pre- and post-operative HRQoL measures for HNPGL. A difference in HRQoL between patients who were operated on and patients who were conservatively treated could not be shown in cross-sectional studies [33, 35, 43]. However, post hoc power calculation revealed insufficient power to detect these differences between groups [35]. Briner et al. (1998) showed that most patients resume work (72%), have a normal social life (69%) and feel like before surgery (56%) within 6 months postoperative; after 2 year this has increased to respectively 97%, 97%, and 75% [41].

**Table 3:** Mean and median HRQoL measured with EORTC QLQ-C30 in HNPGL patients

Study	Galland-Girodet 2014 ^a^	Cao 2018 ^a^	De Bresser 2025 ^b^	De Bresser 2025 ^b^	EORTC database Reference values ^ab^ [46]	
Population	HNPGL (*n* = 20)^d^	GJT (*n* = 8)	SDHx variant carrier^e^ (*n* = 84)	Sporadic HNPGL^f^ (*n* = 16)	Head and neck cancer age 50–59	General population
Intervention	RTx (*n* = 9), RTx+ Surgery (*n* = 11)	Proton therapy	N/A	N/A		
Timing	102 (12–228) mo after therapy ^a^	17.6 (2–23) mo after therapy^a^	Cross-sectional	Cross-sectional		
Global health status (QoL)	58 ± 24^a^	66.6 (50–100)^a^	83.3 [75–100]^b^	75 [67–90]^b^	63.6 ± 22.9^a^ 66.7 [50-83.3]^b^	71.2 ± 22.4^a^ 75 [58.3–83.3]^b^
Physical functioning	76.1 ± 22.4^a^	84.1 (46.7–100)^a^	100 [93–100]^b^	93.3 [73–100]^b^	83.3 ± 19.1^a^ 93.3 [73.3–100]^b^	89.8 ± 16.2^a^ 100 [86.7–100]^b^
Role functioning	77.2 ± 29.5^a^	74.9 (33.3–100)^a^	100 [83–100]^b^	100 [71–100]^b^	79.5 ± 28.4^a^ 100 [66.7–100]^b^	84.7 ± 25.4^a^ 100 [66.7–100]^b^
Emotional functioning	65.4 ± 36.5^a^	78.1 (41.6–100)^a^	91.7 [67–100]^b^	91.7 [67–100]^b^	70.5 ± 25.1 ^a^ 75 [58.3–91.7] ^b^	76.3 ± 22.8^a^ 83.3 [66.7–100]^b^
Cognitive functioning	70.2 ± 29.2^a^	74.9 (33.3–100)^a^	100 [83–100]^b^	100 [71–100]^b^	86.4 ± 20.3 ^a^ 100 [83.3–100] ^b^	86.1 ± 20^a^ 100 [83.3–100]^b^
Social functioning	60.5 ± 36.5^a^	83.3 (50–100)^a^	100 [83–100]^b^	100 [67–100]^b^	81.7 ± 25.4 ^a^ 100 [66.7–100] ^b^	87.5 ± 22.9^a^ 100 [83.3–100]^b^

(…), Range; […], Interquartile range; ±, Standard deviation; CBT, Carotid Body Tumor; GJT, Jugulotympanic paraganglioma; HNPGL, Head and Neck Paraganglioma not specified; mo, months; N/A, not available.

^a^Mean value

^b^Median value

^d^20 questionnaires from 30 patients with 42 HNPGL: GJT (*n* = 23), CBT (*n* = 10), vagal paraganglioma (*n* = 9)

^e^Of these patients 40/84 (48%) were presymptomatic (i.e., no HNPGL) SDHx variant carriers

^f^Of these patients 7/16 (44%) had no detectable paraganglioma after surgery

### Effects of malignancy, symptomatology, and age on HRQoL

Beyond tumor location and treatment modality, several additional factors influenced the HRQoL of patients. A higher number of HNPGLs is significantly associated with greater feelings of depression [35] and general fatigue [33]. Furthermore, a higher number of HNPGLs had a negative effect on physical functioning and general health perception [35]. Patients with a history of surgically treated pheochromocytomas or extra-adrenal paragangliomas were not found to have an altered QoL compared with the rest of the patient group [33, 35]. Patients with malignant paraganglioma reported significantly more mental fatigue and had a reduced score on the general health perception subscale; other subscales were not affected [35].

Regarding symptomatology, most local complaints (e.g., hearing loss, tinnitus, swallowing difficulties) did not significantly affect SF-36 subscales, apart from dysphonia. Dysphonia significantly impaired activity and mental fatigue (MFI-20), as well as physical functioning and general health perception (SF-36) [33]. Systemic complaints due to excessive production of catecholamines (e.g., palpitations, perspiration, panic attacks, vertigo, headaches) was associated with significantly increased scores on the HADS and MFI-20 and impaired scores on SF-36 on more than eight out of 16 subscales [35, 43].

As anticipated, age negatively impacted the SF-36 domain physical functioning [33, 35]. While Havekes et al. (2008) reported significant effects of age on bodily pain and emotional role limitations, Van Hulsteijn et al. (2014) did not replicate these findings. Females had statistically significant increased anxiety scores, impairment in physical mobility, and experienced more pain [33, 35]. Finally, longitudinal data from Van Hulsteijn et al. (2013) revealed no significant deterioration in HADS or MFI-20 scores over a five-year period, except for a decline in the activity subdomain [35].

## Discussion

This is the first review assessing HRQoL in patients with HNPGL. For these patients, HRQoL has most frequently been measured using either the SF-36 or EORTC QLQ-C30, supplemented by tools assessing fatigue, anxiety, depression, sleep quality, and cranial nerve-related dysfunction. These results show that HNPGL negatively affects HRQoL across several domains, even in untreated patients. Specific subgroups, including those with CBT, multiple tumors, or dysphonia, experience disproportionately lower HRQoL. Fatigue and sleep-related complaints are prevalent, and cranial nerve dysfunction (especially dysphonia) was shown to have a significant impact on functioning. In contrast, tinnitus, hearing loss, other local symptoms, and presymptomatic SDHx genetic variants were not consistently associated with reduced HRQoL. Nevertheless, it is conceivable that certain presymptomatic patients with genetic variants that are associated with a higher risk of metastasis or multiple tumors may experience more anxiety or loss of HRQoL. Notably, although patients may have worse HRQoL current evidence does not clearly establish that surgical or radiotherapeutic interventions independently worsen HRQoL. Pre- and post-treatment comparisons were scarce, underscoring the major gap in prospective longitudinal data to prevent reverse causation bias.

### Evaluation of questionnaires

There are no studies comparing the use of the SF-36 to the EORTC QLQ-C30 in patients with HNPGL. The SF-36 has globally been one of the most used generic instruments for HRQoL assessment in adults especially in large varied cohorts [47]. The EORTC QLQ-C30 similarly provides a generic assessment of a patients’ HRQoL with a high correlation to equivalent SF-13 domains, however it has been specifically designed and validated for its use on cancer patients [48]. Consequently, where the SF-36 addresses no other symptoms than general pain and fatigue, the EORTC QLQ-C30 also includes sleep, financial difficulties, nausea/vomiting, dyspnea, appetite loss, constipation, and diarrhea [48]. In addition to these generic questionnaires, the EORTC has provided additional modules such as the QLQ-HN35 and newly developed QLQ-HN43 to capture the problems that patients with head and neck cancer experience [49]. While these may improve its applicability to HNPGL by including certain specific symptoms (e.g., dysphonia), effects resulting from skull base or endocrinological involvement are omitted, due to it primarily being developed based on interviews with patients with cancer in the oral cavity (29%), oropharynx (24%), and larynx (24%) [50].

Inclusion of additional HNPGL specific symptoms or qualia could improve the sensitivity to disease or treatment specific burdens even though these might not impact the resulting scores of the generic HRQoL measures. In other words, although some patients with HNPGLs may experience considerable problems owing to their HNPGL(s) and/or its treatment, they have successfully adjusted to living with their problems and thus score comparable to reference values [51]. Nevertheless, in optimizing the care for these patients and evaluation of various management strategies, a more sensitive HNPGL disease-specific questionnaire could be instrumentally valuable [19, 38, 42].

### Comprehensiveness and applicability of evidence

This review, although providing a comprehensive overview of current literature, underscores the limited scope of existing studies. Although studies that include quantifiable HRQoL data as a secondary outcome measure while not mentioning this in their title or abstract were not identified, due to the use of citation searching it is unlikely that relevant studies were missed. Of the included studies, many lacked a robust study design for reliable interpretation of HRQoL, such as prospective data collection, control groups, or adequate sample sizes. Additionally, since most studies used generic questionnaires, sensitivity to changes in HRQoL may not be precise enough to differentiate between clinically significant or relevant differences after for instance a certain therapy.

Furthermore, the lack of stratification in certain studies might have resulted in both an over- or underestimation of HRQoL when, for instance, a considerable proportion of the cohort were asymptomatic or when patients received radiotherapy when conservative management could have had sufficed. One study compared sporadic HNPGL to SDHx genetic variant carriers with (52%) or without (48%) HNPGL [43]. The inclusion of presymptomatic genetic variant carriers is valuable to assess the impact of long-term active surveillance, however this cannot be examined using the current stratification.

Even though this review adhered to PRISMA guidelines and performed a comprehensive search, potential biases include the reliance on published data which might have caused underrepresentation of negative or inconclusive findings in for example the effect of radiotherapy on HRQoL. The heterogeneity of the included studies precluded the intended quantitative synthesis of the results limiting its ability to draw statistically significant conclusions. Most studies were conducted by west European research groups, which may not accurately reflect the experience of receiving care for HNPGL in other regions with differing healthcare systems and cultural biases.

### Implications and future research

Compared to a similar mostly benign slow growing tumor in the skull base, patients with HNPGL seems to score comparable to slightly more favorable than vestibular Schwannoma patients [45, 52] and on par with the average head and neck cancer patients aged 50–59 year [46].

Although clinicians should recognize the significant impact of HNPGL on HRQoL, this budding line of research in HRQoL may not unequivocally impact current treatment decision yet, however impending data on the consequence of different interventions on HRQoL may. Qualitative research on the psycho-social impact of living with HNPGL is necessary to optimize after care and support systems. It is foreseeable that smaller HNPGLs imposes a limited impact on HRQoL for most patients, however for those that are affected proper identification and understanding of their strife is necessary. Thus, future qualitative research should not only focus on the average patient, instead on those who experience greater than average loss in HRQoL.

To guide future research, this review identified four aims. Firstly, because of the limited sensitivity of generic HRQoL questionnaires, development and validation of a disease specific HRQoL instrument is desired. Secondly, studies should aim to generate prospective longitudinal data before and after treatment. Thirdly, HRQoL outcomes should be stratified by tumor classification, treatment modality, and genetic variant to identify subgroups with distinct needs. Fourthly, qualitative research on the long-term psycho-social impact of living with HNPGL is necessary to optimize after care and support systems.

This review provides a comprehensive critical appraisal of the currently spares evidence on quality of life in patients with head and neck paraganglioma, demonstrating significant impairment and recognizing a range of factors influencing this outcome. By addressing the here identified research gaps and heeding the toils of these patients in clinic, patient-centered care might increase the well-being of these unique patient population by understanding the impact of various treatment options on their life’s.

## Electronic Supplementary Material

Below is the link to the electronic supplementary material.

Supplementary Material 1
