# Supplementary material for: Mapping the neglected topic in head and neck paraganglioma research: a PRISMA scoping review on quality of life
Source: Qual Life Res. 2026 Mar 13;35(4):97. doi: 10.1007/s11136-026-04218-8 (PMC12987911; doi:10.1007/s11136-026-04218-8)
Supplement: Supplementary file 1 — Supplementary Material 1 [file 11136_2026_4218_MOESM1_ESM.docx]

# Supplement 1: Search

| Search | Query **PubMed 24-4-25** | Results |
| --- | --- | --- |
| 1  **HNPGL** | ( ((Paraganglio*[Title/Abstract] OR paraganglioma*[Title/Abstract] OR glomus*[Title/Abstract] OR chemodecto*[Title/Abstract] OR PGL[Title/Abstract] OR PPGL*[Title/Abstract]) AND (head[Title/Abstract] OR neck[Title/Abstract] OR cervical[Title/Abstract] OR caroti*[Title/Abstract] OR tympani*[Title/Abstract] OR tympano*[Title/Abstract] OR temporal*[Title/Abstract] OR jugul*[Title/Abstract] OR middle ear[Title/Abstract] OR vagal*[Title/Abstract] OR larynx*[Title/Abstract] OR sino*[Title/Abstract] OR nasal*[Title/Abstract]))  OR (caroti*[Title/Abstract] AND body[Title/Abstract] AND (tumor*[Title/Abstract] OR tumour*[Title/Abstract]))  OR HNPGL*[Title/Abstract]  OR "Paraganglioma, Extra-Adrenal"[MeSH Terms] ) | 8634 |
| 2  **QoL OR PROMs** | ("Patient Health Questionnaire"[Mesh] OR "Quality of Life"[Mesh] OR "Health Status"[Mesh] OR "Surveys and Questionnaires"[Mesh]  OR quality of life[Title/Abstract] OR QoL[Title/Abstract] OR HRQoL[Title/Abstract] OR HRQL[Title/Abstract] OR health status[Title/Abstract] OR health index[Title/Abstract] OR health indices[Title/Abstract] OR health profile*[Title/Abstract] OR well-being[Title/Abstract] OR questionnaire*[Title/Abstract] OR survey*[Title/Abstract] OR instrument*[Title/Abstract]  OR ( (patient*[Title/Abstract] OR self[Title/Abstract] OR child*[Title/Abstract] OR parent*[Title/Abstract] OR carer[Title/Abstract] OR caretaker*[Title/Abstract] OR proxy[Title/Abstract] OR subjective[Title/Abstract]) AND (report*[Title/Abstract] OR rate*[Title/Abstract] OR assess*[Title/Abstract] OR measur*[Title/Abstract]) AND (function*[Title/Abstract] OR outcome*[Title/Abstract] OR disabilit*[Title/Abstract] OR index[Title/Abstract] OR well-being[Title/Abstract])) ) | 5.008.880 |
| 3  **Specific questionnaires** | ("EuroQol 5D-5L"[Title/Abstract] OR "EQ-5D-5L"[Title/Abstract] OR "EQ-5D"[Title/Abstract] OR "EORTC QLQ-C30"[Title/Abstract] OR "EORTC QLQ"[Title/Abstract] OR "Modified Fatigue Impact Scale"[Title/Abstract] OR "MFIS"[Title/Abstract] OR "Multidimensional Fatigue Index"[Title/Abstract] OR "Short Questionnaire to Assess Health-enhancing physical activity"[Title/Abstract] OR "SQUASH"[Title/Abstract] OR "Cancer Worry Scale"[Title/Abstract] OR "Hospital Anxiety and Depression Scale"[Title/Abstract] OR "HADS"[Title/Abstract] OR "Epworth Sleepiness Scale"[Title/Abstract] OR "PROMIS"[Title/Abstract] OR "PROMIS-10"[Title/Abstract] OR "VRQoL"[Title/Abstract] OR "Voice Handicap Index"[Title/Abstract] OR "Eating Assessment Tool"[Title/Abstract] OR "EAT-10"[Title/Abstract] OR "Voice-Related Quality of Life"[Title/Abstract] OR "VHI"[Title/Abstract] OR "HHIA"[Title/Abstract] OR "DHI"[Title/Abstract] OR "THI"[Title/Abstract] OR "Hearing Handicap Inventory"[Title/Abstract] OR "Dizziness Handicap Inventory"[Title/Abstract] OR "Tinnitus Handicap Inventory"[Title/Abstract] OR "Short Form 36"[Title/Abstract] OR "Short Form 12"[Title/Abstract] OR "SF-36"[Title/Abstract] OR "SF-12"[Title/Abstract] OR "SF36"[Title/Abstract] OR "SF12"[Title/Abstract] OR "RAND-36"[Title/Abstract] OR ((Short Form*[Title/Abstract] OR RAND[Title/Abstract]) AND (36[Title/Abstract] OR 12[Title/Abstract]))) | 106.463 |
| 4 | #1 AND (#2 OR #3) | 818 |

| Search | Query **Embase 24-4-25** | Results |
| --- | --- | --- |
| 1  **HNPGL** | ('paraganglioma':.ti,ab,kf OR 'glomus':.ti,ab,kf OR 'chemodectoma':.ti,ab,kf OR 'PGL':.ti,ab,kf OR 'PPGL':.ti,ab,kf) AND  ('head':.ti,ab,kf OR 'neck':.ti,ab,kf OR 'cervical':.ti,ab,kf OR 'carotid*':.ti,ab,kf OR 'tympanic*':.ti,ab,kf OR  'temporal*':.ti,ab,kf OR 'jugular*':.ti,ab,kf OR 'middle ear':.ti,ab,kf OR 'vagal*':.ti,ab,kf OR 'larynx*':.ti,ab,kf OR  'sino*':.ti,ab,kf OR 'nasal*':.ti,ab,kf) OR  ('carotid*':.ti,ab,kf AND 'body':.ti,ab,kf AND ('tumor*':.ti,ab,kf OR 'tumour*':.ti,ab,kf)) OR  'HNPGL*':.ti,ab,kf OR 'Paraganglioma, Extra-Adrenal':exp | 8547 |
| 2  **QoL OR PROMs** | ('quality of life': or 'QoL': or 'HRQoL': or 'HRQL': or 'life quality': or 'health index': or 'health indices': or 'health profile*': or 'health status': or (('patient*': or 'self': or 'child*': or 'parent*': or 'carer': or 'caretaker*': or 'proxy': or 'subjective':) and ('report': or 'reported': or 'reporting': or 'rated': or 'rating*': or 'assessment*': or 'measure*':) and ('function*': or 'outcome*': or 'disable*': or 'disabilit*': or 'index': or 'indices': or 'well-being': or 'instrument*': or 'measure*': or 'questionnaire*': or 'survey*':))).ti,ab,kf. | 4.991.399 |
| 3  **Specific questionnaires** | ('EuroQol 5D-5L':.ti,ab,kf OR 'EQ-5D-5L':.ti,ab,kf OR 'EORTC QLQ-C30':.ti,ab,kf OR  'Modified Fatigue Impact Scale':.ti,ab,kf OR 'MFIS*':.ti,ab,kf OR  'Short Questionnaire to Assess Health-enhancing physical activity':.ti,ab,kf OR  'SQUASH*':.ti,ab,kf OR 'Cancer Worry Scale':.ti,ab,kf OR  'Hospital Anxiety and Depression Scale':.ti,ab,kf OR  'HADS*':.ti,ab,kf OR  (('Short Form*':.ti,ab,kf OR 'RAND':.ti,ab,kf) AND ('36':.ti,ab,kf OR '12':.ti,ab,kf)) OR  'SF-12':.ti,ab,kf OR  'SF-36':.ti,ab,kf OR  'SF36':.ti,ab,kf OR  'SF12':.ti,ab,kf OR  'RAND-36':.ti,ab,kf OR  'PROMIS*':.ti,ab,kf OR  'Epworth Sleepiness Scale':.ti,ab,kf OR  'Multidimensional Fatigue Index':.ti,ab,kf) | 2.008.118 |
| 4 | #1 AND (#2 OR #3) | 1248 |
| 5 | Limit #4 to ‘’remove medline records’’ AND ‘’Human” | 586 |
| 6 | Limit #5 to conference abstract status | 421 |
| 7 | #5 NOT #6 | 165 |

# Supplement 2: PRISMA-ScR Checklist

| **SECTION** | **ITEM** | **PRISMA-ScR CHECKLIST ITEM** | **REPORTED ON PAGE #** |
| --- | --- | --- | --- |
| **TITLE** | | | |
| Title | 1 | Identify the report as a scoping review. | 1 |
| **ABSTRACT** | | | |
| Structured summary | 2 | Provide a structured summary that includes (as applicable): background, objectives, eligibility criteria, sources of evidence, charting methods, results, and conclusions that relate to the review questions and objectives. | 2 |
| **INTRODUCTION** | | | |
| Rationale | 3 | Describe the rationale for the review in the context of what is already known. Explain why the review questions/objectives lend themselves to a scoping review approach. | 4 |
| Objectives | 4 | Provide an explicit statement of the questions and objectives being addressed with reference to their key elements (e.g., population or participants, concepts, and context) or other relevant key elements used to conceptualize the review questions and/or objectives. | 4 |
| **METHODS** | | | |
| Protocol and registration | 5 | Indicate whether a review protocol exists; state if and where it can be accessed (e.g., a Web address); and if available, provide registration information, including the registration number. | 5 |
| Eligibility criteria | 6 | Specify characteristics of the sources of evidence used as eligibility criteria (e.g., years considered, language, and publication status), and provide a rationale. | 5 |
| Information sources* | 7 | Describe all information sources in the search (e.g., databases with dates of coverage and contact with authors to identify additional sources), as well as the date the most recent search was executed. | 5 |
| Search | 8 | Present the full electronic search strategy for at least 1 database, including any limits used, such that it could be repeated. | 16-17 |
| Selection of sources of evidence† | 9 | State the process for selecting sources of evidence (i.e., screening and eligibility) included in the scoping review. | 5 |
| Data charting process‡ | 10 | Describe the methods of charting data from the included sources of evidence (e.g., calibrated forms or forms that have been tested by the team before their use, and whether data charting was done independently or in duplicate) and any processes for obtaining and confirming data from investigators. | 5 |
| Data items | 11 | List and define all variables for which data were sought and any assumptions and simplifications made. | 5 |
| Critical appraisal of individual sources of evidence§ | 12 | If done, provide a rationale for conducting a critical appraisal of included sources of evidence; describe the methods used and how this information was used in any data synthesis (if appropriate). | 19 |
| Synthesis of results | 13 | Describe the methods of handling and summarizing the data that were charted. | 5 |
| **RESULTS** | | | |
| Selection of sources of evidence | 14 | Give numbers of sources of evidence screened, assessed for eligibility, and included in the review, with reasons for exclusions at each stage, ideally using a flow diagram. | 6 |
| Characteristics of sources of evidence | 15 | For each source of evidence, present characteristics for which data were charted and provide the citations. | 7 |
| Critical appraisal within sources of evidence | 16 | If done, present data on critical appraisal of included sources of evidence (see item 12). | 19 |
| Results of individual sources of evidence | 17 | For each included source of evidence, present the relevant data that were charted that relate to the review questions and objectives. | 7-10 |
| Synthesis of results | 18 | Summarize and/or present the charting results as they relate to the review questions and objectives. | 7-10 |
| **DISCUSSION** | | | |
| Summary of evidence | 19 | Summarize the main results (including an overview of concepts, themes, and types of evidence available), link to the review questions and objectives, and consider the relevance to key groups. | 11 |
| Limitations | 20 | Discuss the limitations of the scoping review process. | 11-12 |
| Conclusions | 21 | Provide a general interpretation of the results with respect to the review questions and objectives, as well as potential implications and/or next steps. | 12 |
| **FUNDING** | | | |
| Funding | 22 | Describe sources of funding for the included sources of evidence, as well as sources of funding for the scoping review. Describe the role of the funders of the scoping review. | 15 |

# Supplement 3: Joanna Briggs Institute’s analytical cross sectional studies critical appraisal tool

Moola S, Munn Z, Tufanaru C, Aromataris E, Sears K, Sfetcu R, Currie M, Qureshi R, Mattis P, Lisy K, Mu P-F. Chapter 7: Systematic reviews of etiology and risk . In: Aromataris E, Munn Z (Editors). JBI Manual for Evidence Synthesis. JBI, 2020. Available from https://synthesismanual.jbi.global

| Study | Year | Q1 | Q2 | Q3 | Q4 | Q5 | Q6 | Q7 | Q8 |
| --- | --- | --- | --- | --- | --- | --- | --- | --- | --- |
| Brinner et al. | 1998 | Yes | Yes | Yes | Yes | No | No | No | No |
| Kollert et al. | 2006 | Yes | No | Yes | Yes | No | No | Yes | No |
| Henzel et al. | 2007 | Yes | Yes | Yes | Yes | Unclear | No | Yes | Yes |
| Havekes et al. | 2008 | Yes | Yes | Yes | Yes | Yes | Yes | Yes | Yes |
| Havekes et al. | 2012 | Yes | Yes | Yes | Yes | Yes | Yes | Yes | Yes |
| Van Hulsteijn et al. | 2013 | Yes | Yes | Yes | Yes | Yes | Yes | Yes | Yes |
| Van Hulsteijn et al. | 2014 | Yes | Yes | Yes | Yes | Yes | Yes | Yes | Yes |
| Galland-Girodet et al. | 2014 | Yes | Yes | Yes | Yes | Yes | Yes | Yes | Yes |
| Cao et al. | 2018 | Yes | Yes | Yes | Yes | Unclear | No | Yes | Yes |
| Garcia-Alva et al. | 2019 | Yes | Yes | Yes | Yes | Yes | Yes | Yes | Yes |
| Patel et al. | 2019 | Yes | Yes | Yes | Yes | Yes | Yes | Yes | Yes |
| Ehret et al. | 2020 | Yes | Yes | Yes | Yes | Yes | Yes | Yes | Yes |
| Hebb et al. | 2020 | Yes | No | Yes | Yes | Unclear | No | Yes | No |
| De Bresser et al. | 2025 | Yes | No | Yes | Yes | Unclear | No | Yes | Yes |

**Q1. Were the criteria for inclusion in the sample clearly defined?**

The authors should provide clear inclusion and exclusion criteria that they developed prior to recruitment of the study participants. The inclusion/exclusion criteria should be specified (e.g., risk, stage of disease progression) with sufficient detail and all the necessary information critical to the study.

**Q2. Were the study subjects and the setting described in detail?**

The study sample should be described in sufficient detail so that other researchers can determine if it is comparable to the population of interest to them. The authors should provide a clear description of the population from which the study participants were selected or recruited, including demographics, location, and time period.

**Q3. Was the exposure measured in a valid and reliable way?**

The study should clearly describe the method of measurement of exposure. Assessing validity requires that a 'gold standard' is available to which the measure can be compared. The validity of exposure measurement usually relates to whether a current measure is appropriate or whether a measure of past exposure is needed.

Reliability refers to the processes included in an epidemiological study to check repeatability of measurements of the exposures. These usually include intra-observer reliability and inter-observer reliability.

**Q4. Were objective, standard criteria used for measurement of the condition?**

It is useful to determine if patients were included in the study based on either a specified diagnosis or definition. This is more likely to decrease the risk of bias. Characteristics are another useful approach to matching groups, and studies that did not use specified diagnostic methods or definitions should provide evidence on matching by key characteristics

**Q5. Were confounding factors identified?**

Confounding has occurred where the estimated intervention exposure effect is biased by the presence of some difference between the comparison groups (apart from the exposure investigated/of interest). Typical confounders include baseline characteristics, prognostic factors, or concomitant exposures (e.g. smoking). A confounder is a difference between the comparison groups and it influences the direction of the study results. A high quality study at the level of cohort design will identify the potential confounders and measure them (where possible). This is difficult for studies where behavioral, attitudinal or lifestyle factors may impact on the results.

**Q6. Were strategies to deal with confounding factors stated?**

Strategies to deal with effects of confounding factors may be dealt within the study design or in data analysis. By matching or stratifying sampling of participants, effects of confounding factors can be adjusted for. When dealing with adjustment in data analysis, assess the statistics used in the study. Most will be some form of multivariate regression analysis to account for the confounding factors measured.

**Q7. Were the outcomes measured in a valid and reliable way?**

Read the methods section of the paper. If for e.g. lung cancer is assessed based on existing definitions or diagnostic criteria, then the answer to this question is likely to be yes. If lung cancer is assessed using observer reported, or self-reported scales, the risk of over- or under-reporting is increased, and objectivity is compromised. Importantly, determine if the measurement tools used were validated instruments as this has a significant impact on outcome assessment validity.

Having established the objectivity of the outcome measurement (e.g. lung cancer) instrument, it’s important to establish how the measurement was conducted. Were those involved in collecting data trained or educated in the use of the instrument/s? (e.g. radiographers). If there was more than one data collector, were they similar in terms of level of education, clinical or research experience, or level of responsibility in the piece of research being appraised?

**Q8. Was appropriate statistical analysis used?**

As with any consideration of statistical analysis, consideration should be given to whether there was a more appropriate alternate statistical method that could have been used. The methods section should be detailed enough for reviewers to identify which analytical techniques were used (in particular, regression or stratification) and how specific confounders were measured.

For studies utilizing regression analysis, it is useful to identify if the study identified which variables were included and how they related to the outcome. If stratification was the analytical approach used, were the strata of analysis defined by the specified variables? Additionally, it is also important to assess the appropriateness of the analytical strategy in terms of the assumptions associated with the approach as differing methods of analysis are based on differing assumptions about the data and how it will respond.
